# Selexipag-based triple combination therapy improves prognosis in Chinese pulmonary arterial hypertension patients

**DOI:** 10.3389/fcvm.2022.991586

**Published:** 2022-09-20

**Authors:** Xiaopei Cui, Weida Lu, Deyuan Zhang, Liangyi Qie, Haijun Li, Xiao Li, Hui Liu, Qiushang Ji

**Affiliations:** ^1^Key Laboratory of Cardiovascular Proteomics of Shandong Province, Department of Geriatric Medicine, Qilu Hospital, Cheeloo College of Medicine, Shandong University, Jinan, Shandong, China; ^2^Huantai County People’s Hospital, Huantai, Shandong, China; ^3^Department of General Practice, Qilu Hospital, Cheeloo College of Medicine, Shandong University, Jinan, Shandong, China; ^4^Department of Cardiology, Qilu Hospital, Cheeloo College of Medicine, Shandong University, Jinan, Shandong, China

**Keywords:** pulmonary arterial hypertension, triple combination, selexipag, tolerability, risk assessment

## Abstract

**Aim:**

Selexipag is an oral selective prostacyclin receptor agonist approved for treatment of patients with pulmonary arterial hypertension (PAH). In the present study, we aim to assess the safety and efficacy of selexipag in triple combination therapy with endothelial receptor antagonists (ERAs) and PDE5is for Chinese PAH patients.

**Methods and results:**

A single center retrospective study was performed on group 1 PAH patients (*n* = 68) initiating triple combination therapy with selexipag from 1 February 2020 to 31 August 2021 in Qilu Hospital of Shandong University (Shandong, China). Adolescents, children, and PAH patients with unrepaired congenital heart disease were excluded. The French pulmonary hypertension network (FPHN) non-invasive risk assessment, echocardiogram parameters, and clinical data, including tolerability, safety, and death/hospitalization events associated with PAH, were collected. Of the 68 patients, 31 (45.6%) patients had tolerable side effects while only a single patient discontinued selexipag due to severe diarrhea. In the analysis of the efficacy set of 62 patients, the median selexipag treatment time from selexipag initiation to last risk assessment was 27 (21, 33) weeks. Compared to baseline parameters, the percentage of WHO FC III/IV decreased from 77.4% (48) to 24.2% (15) (*p* = 0.000), median 6-min walk distance (6MWD) increased 82 m [from 398 (318, 450) to 480 (420, 506) m; *p* = 0.000], and NT-proBNP levels decreased from 1,216 (329, 2,159) to 455 (134, 1,678) pg/mL (*p* = 0.007). Patients who improved to three low-risk criteria increased from 9.7 to 38.7%. Right ventricular diameter (RV) diameter also decreased and was accompanied by an improved tricuspid annular plane systolic excursion (TAPSE). Patients transitioning from subcutaneous treprostinil to selexipag continued to show improvements in WHO FC, 6MWD (404 ± 94 vs. 383 ± 127 m) and NT-proBNP levels (2,319 ± 2,448 vs. 2,987 ± 3,770 pg/mL). Finally, the 1-year event free survival rate was 96.7% for patients initiating the triple combination therapy within 3 years of PAH diagnosis.

**Conclusion:**

Triple combination therapy with selexipag was safe and effective in Chinese PAH patients, which was confirmed by acceptable tolerability, and improved exercise capacity, right heart function, risk assessment, and prognosis.

## Introduction

Pulmonary arterial hypertension (PAH) is a devastating disease characterized by progressive pulmonary artery remodeling and right heart failure with high mortality (1). In the past decades, PAH-specific therapy has greatly improved the survival rate of patients with this disease (2, 3). The combination of drugs targeting endothelin-1, nitric oxide, and prostacyclin (IP) have further improved patient response (4, 5). To date, the rationale for PAH treatment strategy is based on disease severity assessed with multi-parametric risk stratification approaches to help PAH patients achieve and maintain a low-risk status (6, 7).

Selexipag, an orally available selective IP receptor agonist, is an approved therapy for PAH (5). In the GRIPHON trial, patients receiving selexipag either as monotherapy or in addition to endothelial receptor antagonists (ERAs) and/or phosphodiesterase type 5 inhibitors (PDE5is) showed a 40% risk reduction in clinical worsening events (5). As a randomized controlled trial, the GRIPHON trial established the efficacy and safety of selexipag, but real-world evidence has also been important in providing complementary data for routine clinical practice. For instance, in GRIPHON trial, 14.3% patients discontinued selexipag treatment due to adverse event, most frequently reported was headache. However, in the SPHERE Registry from the United States the most reported side effect was gastrointestinal disorders, the discontinuation rate due to adverse event related to selexipag was 7.2% and the titration of selexipag is more complicated and proceeds more slowly in general practice (8). Another real-world study carried out in Germany intriguingly indicated that patients with side effects during titration responded better to selexipag treatment (9).

To date, data are limited for the treatment of Asian PAH patients with selexipag. Two hundred and twenty-three Asian patients were included in the GRIPHON trial with half of them treated with selexipag and the results did not show any benefit for this subgroup (5). Although the JAPIC trial carried out in Japan demonstrated that selexipag is effective in Japanese PAH patients (10), efficacy in Chinese PAH patients has not yet been rigorously evaluated.

Here, we performed a retrospective study in Chinese PAH patients treated with selexipag to investigate the efficacy and safety of the drug in general practice.

## Materials and methods

### Study design and patient enrollment

**FIGURE 1:**
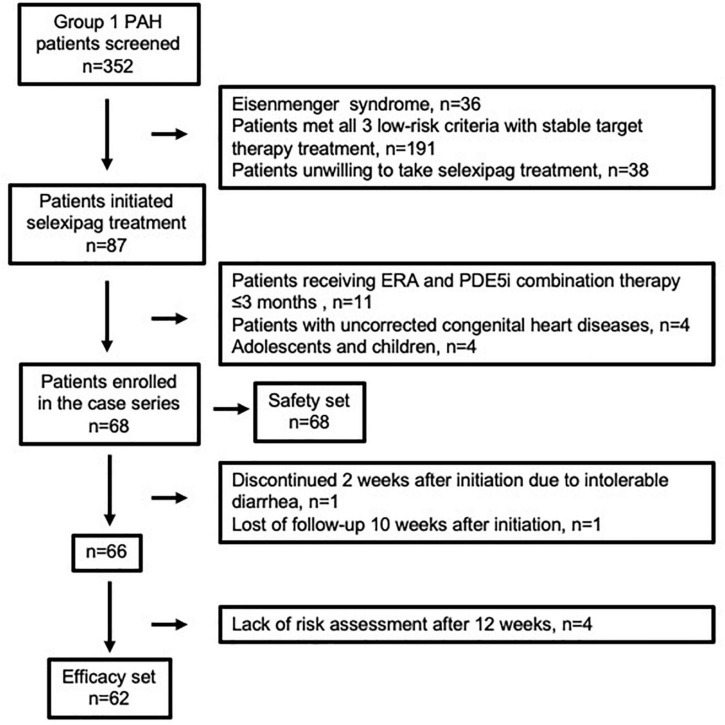
Enrollment flow chart for safety and efficacy set in the present case series.

This single center, retrospective uncontrolled study was carried out in Qilu Hospital of Shandong University in northern China. Group 1 PAH patients diagnosed by right heart catheterization, including idiopathic PAH (IPAH), heritable PAH (HPAH), post-operative PAH associated with congenital heart disease (post-operative CHD-PAH), PAH associated with connective tissue diseases (CTD-PAH), and PAH associated with HIV infection were screened. Those who has Eisenmenger syndrome, met all three low-risk criteria with stable target therapy treatment or unwilling to take selexipag treatment were excluded from selexipag initiation. Those who began triple treatment which included selexipag, ERAs and PDE5is from 1 February 2020 to 31 August 2021, were recruited for the study. For sequential addition of selexipag to ERA and PDE5i, only patients who had already received at least 3 months stable treatment with ERA and PDE5i were included. Adolescents, children and patients with PAH related to uncorrected congenital heart disease (having treat-to-repair therapy) who took selexipag were excluded from the current analysis. The flow chart of enrollment was shown in Figure 1. All patients were recommended to undergo clinical evaluation every 3 months. The cutoff date for follow-up data collection was 31 December 2021. For the analysis, the safety set included all patients (*n* = 68) taking at least one dose of selexipag, while the efficacy set included patients (*n* = 62) taking continuous selexipag treatment over 12 weeks and undergoing at least one risk assessment during follow-up. The study was approved by the Institutional Human Ethics Committee of Qilu Hospital (reference number: 2021072) and written informed consent was exempted.

### Data collection

Non-invasive parameters collected at baseline and follow-up included clinical characteristics, disease characteristics, concurrent/previous PAH therapy within the last 3 months, selexipag titration, transition between selexipag and parenteral prostacyclin, WHO functional class (FC), 6-min walk distance (6MWD), right atrial area (RAA), right ventricular diameter (RV), tricuspid annular plane systolic excursion (TAPSE), hemoglobulin (HGB), and total bilirubin (TB). The clinical data obtained at the last clinical visit on or before the initiation of selexipag treatment were defined as baseline. The WHO FC was determined by an experienced physician, and the same technician performed transthoracic echocardiography during follow-up visits.

### Risk assessment and outcome measures

The French Pulmonary Hypertension Network (FPHN) non-invasive risk assessment strategy using three low-risk criteria is more convenient to use in clinical practice compared to other risk stratification strategies (11–13), and has shown accurate identification of patients with excellent long-term survival (14). In the present study, non-invasive risk assessment was performed using three parameters, including WHO FC, NT-proBNP levels, and 6MWD, as recently suggested (13). Low-risk criteria were defined as follows: (1) WHO FC I or II, (2) NT-proBNP <300 pg/mL, and (3) 6MWD >440 m. The number of low-risk criteria present at baseline and each clinical visit were recorded. For patients with multiple risk assessments before the cutoff date, only the last event was noted for efficacy analysis. Events including hospitalization, death and initiation of parenteral PGIs associated with PAH progression were observed until the cutoff date or until the withdrawal date for patients who discontinued selexipag. For patients with multiple events, only the first event was noted.

### Selexipag dosage titration

Selexipag was initiated in patients at 200 μg twice daily and further up-titration was suggested when side effects subsided. A weekly increase of 200 μg twice daily was recommended, but the 200 μg increment, daily or at longer intervals, was also allowed depending on the tolerance of patients to side effects. The maximum dose allowed was 1,600 μg twice daily. The maintenance dose was defined as the twice daily dose received for the longest duration. Dose levels were defined as follows: low-dose, 200–400 μg twice daily; medium-dose, 600–1,000 μg twice daily; and high-dose, 1,200–1,600 μg twice daily.

### Transition from subcutaneous treprostinil to oral selexipag

The treprostinil dose was down-titrated every 3 days by 2.5 ng/kg/min, and a standard weekly increase in selexipag of 200 μg twice daily was initiated on the same day of the week.

### Selexipag-based initial triple combination

Macitentan 10 mg or ambrisentan 10 mg once daily, and tadalafil 20 mg once daily or sildenafil 25 mg three times daily were initiated on day 1. Selexipag dose titration began on day 8–15.

## Statistical analysis

Analysis was performed with SPSS software, version 13.0 (SPSS Inc., Chicago, IL, USA). Categorical data are presented as counts or percentages. Normal distribution was evaluated using the Kolmogorov–Smirnov test. Continuous variables are presented as the mean with the standard deviation when distributed normally, or otherwise as the median with the interquartile range (IQR). Paired *t*-test, paired rank sum test or the chi-square test were used to compare the differences between baseline and follow-up values where appropriate. Significant differences were defined as *p* < 0.05 (two-tailed test).

## Results

### Baseline characteristics

Sixty-eight patients, 57 (83.8%) females and 11 males (16.2%), were included in the study. The mean age was 31.9 ± 9.5 years. The mean time from PAH diagnosis to initiation of treatment with selexipag was 4.8 ± 4.9 years. All patients had WHO group 1 pulmonary arterial hypertension. The most common type was IPAH (61.8%), followed by post-operative CHD-PAH (25.0%) and CTD-PAH (10.3%). Two patients were also diagnosed as HPAH (2.9%). Fifty patients (73.5%) were already receiving ERA and PDE5i combination treatment (for >3 months), 11 patients (16.2%) were on triple therapy including subcutaneous treprostinil, and seven patients (10.3%) were newly diagnosed and given triple therapy, including ERA, PDE5i, and selexipag, at diagnosis. The WHO FCs at baseline were mainly class III (60.3%) and class II (23.5%).

### Efficacy

**FIGURE 2:**
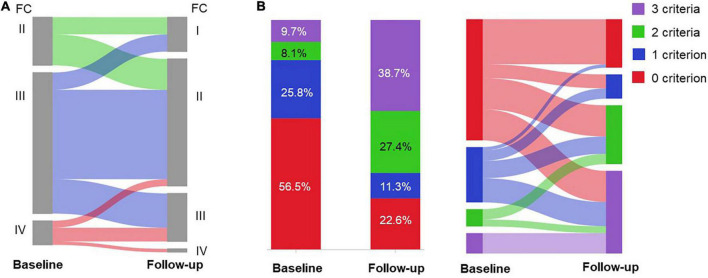
Risk assessment of the patients (*n* = 62) in the efficacy set at baseline and follow-up. Change in **(A)** WHO functional class (FC); and **(B)** the number of low risk criteria after selexipag treatment.

Sixty-two patients were included in the efficacy analysis. The baseline characteristics of the patients are shown in Table 1. The average follow-up visits per patient was 2.6 ± 1.6 times. The median treatment time from initiation to the last risk assessment was 27 (21, 33) weeks. Compared to baseline, the percentage of WHO FC III/IV decreased from 77.4% (48) to 24.2% (15) (*p* = 0.000; Figure 2A). The median 6MWD increased 82 m [from 398 (318, 450) to 480 (420, 506) m; *p* = 0.000], and NT-proBNP levels decreased from 1,216 (329, 2,159) pg/mL to 455 (134, 1,678) pg/mL (*p* = 0.007) (Table 2). Sixty-one patients underwent echocardiographic assessment at baseline and follow-up to assess the effects of selexipag on right heart size and function (Table 2). The RV diameter decreased in treated patients [38 (31, 47) vs. 35 (29, 45) mm; *p* = 0.001], and the RA area showed a decreasing trend from 25 (19, 34) to 21 (15, 33) cm^2^ although this difference was not statistically significant (*p* = 0.113). TAPSE increased from 16 (14, 18) to 18 (15, 20) mm (*p* = 0.002). Finally, total serum bilirubin decreased with treatment (*p* = 0.020).

**TABLE 1:** Baseline characteristics of the case series.

	Total population *n* = 68	Efficacy set *n* = 62
	57 (83.8)	53 (85.5)
Age, mean (SD), years	31.9 (9.5)	31.8 (8.4)
Time from PAH diagnosis to selexipag initiation		
Mean (SD), years	4.8 (4.9)	4.8 (5.0)
PAH etiology, *n* (%)		
IPAH	42 (61.8)	40 (64.5)
Post-operative CHD-PAH	17 (25.0)	15 (24.2)
CTD-PAH	7 (10.3)	6 (9.7)
HPAH	2 (2.9)	1 (1.6)
Combination of selexipag, *n* (%)		
Third add-on to ERA and PDE5i combination	50 (73.5)	46 (74.2)
Transition from subcutaneous treprostinil to selexipag	11 (16.2)	10 (16.1)
Upfront triple combination in newly diagnosed PAH	7 (10.3)	6 (9.7)

Continuous data are expressed as the “mean (SD).” IPAH, idiopathic pulmonary arterial hypertension; CHD-PAH, PAH associated with congenital heart disease; CTD-PAH, PAH associated with connective tissue diseases; HPAH, heritable PAH.

**TABLE 2:** Risk assessment parameters of the efficacy set at baseline and follow-up.

	Baseline	Follow-up	*P*
WHO FC, *n* (%)			<0.0001
I	0 (0.0)	10 (16.1)	
II	14 (22.6)	37 (59.7)	
III	41 (66.1)	14 (22.6)	
IV	7 (11.3)	1 (1.6)	
6MWD, median (IQR), m	398 (318, 450)	480 (420, 506)	<0.0001
NT-proBNP, median (IQR), pg/mL	1,216 (329, 2,159)	455 (134, 1,678)	0.007
RAA, median (IQR), cm^2†^	25 (19, 34)	21 (15, 33)	0.113
RV, median (IQR), mm^†^	38 (31, 47)	35 (29, 45)	0.001
TAPSE, median (IQR), mm^†^	16 (14, 18)	18 (15, 20)	0.002
TBIL, median (IQR), μmol/L^†^	14.1 (10.2, 24.2)	9.6 (11.6, 19.3)	0.020

Continuous data are expressed as the mean (SD) or if not normally distributed as the median (IQR) and compared using paired-*t* test or Wilcoxon matched-pairs signed rank test. Categorical data are compared using Fisher’s exact test. *P < 0.05 between two groups. ^†^Data missing for some subjects. WHO-FC, World Health Organization functional class; 6MWD, six-min walking distance; NT-proBNP, *N*-terminal pro B-type natriuretic peptide; RAA, right atrial area; RV, right ventricle diameter; TAPSE, tricuspid annular plane systolic excursion; TBIL, total bilirubin.

Based on FPHN non-invasive risk assessment, the percentage of patients with all three low-risk criteria increased from 6 (9.7%) to 24 (38.7%), while the proportion of patients with no low-risk criteria decreased from 35 (56.5%) to 14 (22.6%) (*p* < 0.001; Figure 2B). For those 35 patients with no low-risk criteria at baseline, 22 (62.9%) of patients reached at least one low-risk criterion and 25.7% improved to all three low-risk criteria.

Six patients deteriorated due to their PAH during the period from selexipag initiation to the cutoff date (a maximum of 92 weeks). The incidence rate of clinical worsening events was 15.9%/person-year. A single patient died due to PAH progression, while five patients were hospitalized due to declining right heart function related to PAH. One of the hospitalized patients continued selexipag treatment but switched to riociguat from tadalafil, two began to receive subcutaneous injection of treprostinil instead of selexipag, and two patients maintained their course of treatment. Time to clinical worsening was 39 ± 16 weeks and overall 1-year event-free survival was 90.2% (Supplementary Figure 1). Because the disease course correlates with severity, we further compared event-free survival in patients with PAH disease course of >3 years to ≤3 years. One-year event-free survival was 78.7 vs. 96.7%, respectively. Statistical analysis was not carried out due to the small sample size.

### Tolerability, patient disposition, and safety

**FIGURE 3:**
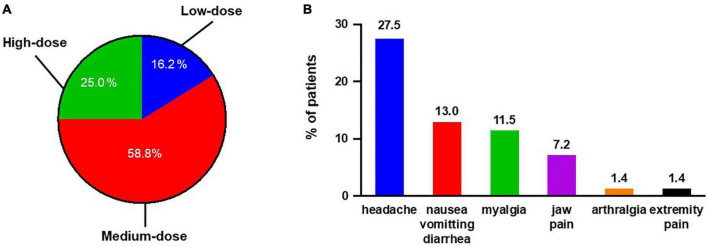
Summary of selexipag usage for all 68 Chinese PAH patients. **(A)** Maintenance dosage of selexipag in Chinese PAH patients: low-dose, 200–400 μg twice daily; medium-dose, 600–1,000 μg twice daily; and high-dose, 1,200–1,600 μg twice daily. **(B)** Side effects reported during maintenance period.

All 68 patients were included in the safety set. The maintenance dose for most patients (*n* = 40; 58.8%) was 600–1,000 μg selexipag twice daily. Seventeen (25.0%) patients received high dose selexipag, while 11 (16.2%) patients received low dose selexipag (Figure 3A). The median time for dose titration was 8 (5, 15) weeks. The side effects reported during the titration phase included headache, nausea/vomiting/diarrhea, myalgia, jaw pain, arthralgia, and extremity pain. All patients experienced at least one side effect during this period. Tolerable side effects occurred in 31 (45.6%) patients during the maintenance phase. The most commonly reported side effects were headache (27.9%), followed by nausea/vomiting/diarrhea (13.2%) and myalgia (11.8%) (Figure 3B). Hepatotoxicity or other unknown side effects were not reported.

Overall, eight patients (11.8%) discontinued selexipag before the cutoff date, and the median time for selexipag treatment was 43 (20, 50) weeks. Two patients transitioned to subcutaneous treprostinil due to unchanged risk status, one patient died due to right heart failure, and four patients were unwilling to continue treatment. Treatment in only one patient was terminated 2 weeks after initiation due to intolerable diarrhea and vomiting with 200 μg twice daily selexipag.

### Subgroups of special interest

**FIGURE 4:**
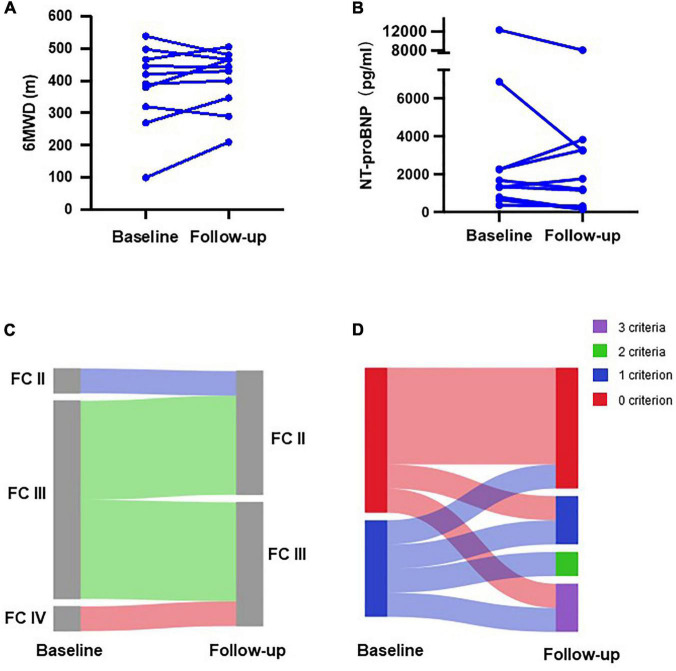
Changes in clinical parameters of patients transitioning from subcutaneous treprostinil to selexipag. Changes in **(A)** 6-min walking distance (6MWD); **(B)** *N*-terminal pro B-type natriuretic peptide (NT-proBNP) level; **(C)** WHO functional class (FC); and **(D)** number of low-risk criteria.

We also separately evaluated patients who transitioned from subcutaneous treprostinil to oral selexipag (*n* = 10). Treatment with treprostinil was terminated in two patients due to intolerable side effects, and in eight patients who were unwilling to continue due to economic burden. The average time to the last risk assessment was 29 ± 15 weeks. Six-minutes walking distance increased from 383 ± 127 to 404 ± 94 m (Figure 4A), and NT-proBNP levels decreased from 2,987 ± 3,770 to 2,319 ± 2,448 pg/mL (Figure 4B). At baseline, only one patient was diagnosed as WHO FC II, while the other nine patients were diagnosed as WHO FC III/IV. All patients showed improvement in the WHO functional class (Figure 4C). At baseline, six patients exhibited zero low-risk criteria, and four patients exhibited one low-risk criterion. After selexipag treatment, two patients reached all three low-risk criteria, two patients improved to meet more low-risk criteria, and four patients remained stable without PAH-associated hospitalization (Figure 4D). Two patients continued to deteriorate and were hospitalized due to right heart failure 9–39 weeks after transition, respectively.

We next focused on the characteristics of patients considered to respond to selexipag treatment. Patients with 0–1 low-risk criteria at baseline and improved in at least two low-risk criteria at follow-up were defined as selexipag responders. All other patients were considered as non-responders, except for patients with all three low-risk criteria at baseline, who were excluded from this analysis. Baseline characteristics between responders and non-responders are summarized in Table 3. Only descriptive analysis was carried out due to the limited sample size. Sex, age, baseline WHO FC, maintenance dosage of selexipag, and total treatment duration at follow-up were comparable between the two groups. Responders included more IPAH patients (81.5 vs. 51.7%), shorter PAH history, lower NT-proBNP levels [947 (334, 1,736) vs. 1,840 (957, 2,588) pg/mL] and smaller RAA [24 (19, 33) vs. 28 (22, 37) cm^2^].

**TABLE 3:** Baseline characteristics of selexipag responder and non-responder.

	Non-responder *n* = 29	Responder *n* = 27
Female, *n* (%)	24 (82.8)	24 (88.9)
Age, mean (SD), years	32.5 (8.2)	31.3 (9.3)
PAH diagnosis to selexipag initiation ≤6 months, n (%)	3 (10.3)	7 (25.9)
PAH etiology, *n* (%)		
IPAH	15 (51.7)	22 (81.5)
CHD-PAH	11 (37.9)	1 (3.7)
CTD-PAH	2 (6.9)	4 (14.8)
HPAH	1 (3.4)	0 (0.0)
Selexipag treatment		
Medium/high-dose	24 (82.8)	22 (81.5)
Duration, median (IQR), weeks	27 (20.5, 34.5)	25 (20, 32)
Upfront triple combination	0 (0)	6 (22.2)
WHO FC III/IV, *n* (%)	24 (82.7)	24 (88.9)
NT-proBNP, median (IQR), pg/mL	1,840 (957, 2,588)	947 (334, 1,736)
RAA, median (IQR), cm^2^	28 (22, 37)^†^	24 (19, 33)
RV, mean (SD), mm	41 (9)^†^	41 (9)
TAPSE, mean (SD), mm	16 (2.7)^†^	17 (3.2)

Continuous data are presented as the “mean (SD) or median (IQR).” ^†^Data missing for one patient. WHO-FC, World Health Organization functional class; NT-proBNP, *N*-terminal pro B-type natriuretic peptide; RAA, right atrial area; RV, right ventricle diameter; TAPSE, tricuspid annular plane systolic excursion.

## Discussion

The GRIPHON trial demonstrated that selexipag targeting the PGI pathway is an effective treatment for PAH. However, the results of the subgroup analysis based on geographic region showed no benefit of the drug for Asian patients (5). Only limited numbers of Chinese patients were included in the GRIPHON trial. Therefore, the efficacy and safety for selexipag in Chinese PAH patients remains unclear. In the present study, we demonstrated that selexipag effectively improves WHO FC, 6MWD, and NT-proBNP, and was accompanied with better risk assessment without unreported side effects. The results indicate that triple combination treatment including selexipag is effective and safe for Chinese PAH patients.

Nowadays, dozens of drugs specific for PAH treatment are commercially available. The treatment strategy for PAH patients, dual combination therapy containing ERA and PDE5i, which evolved in the past decade, is now widely accepted (15, 16). However, approximately 50% of patients treated initially with the combination therapy of ambrisentan and tadalafil for 2 years remain in a medium/high risk status. These results indicate that triple upfront combination therapy with drugs targeting the prostacyclin pathway may be necessary to achieve a more substantially improved prognosis in PAH patients (17). The GRIPHON trial demonstrated that the addition of selexipag in patients treated with ERA and PDE-5i further improved long-term outcomes (18). In the present study, 72.5% patients were already receiving a stable dose of ERAs and PDE5i, but we also noticed that sequential combination with selexipag improved the risk status. This finding is consistent with the results of the GRIPHON study.

Follow-up risk assessment following treatment has been shown to be more reliable in predicting patient survival than the initial risk assessment (6, 13). In the present study, 36 patients still met no low-risk criteria at baseline and 63.86% patients reached at least one low-risk criterion at follow-up. This rate is much higher than that in the COMPARA cohort (48%) and comparable to the French registry (72%) (14). Therefore, selexipag is effective for management of high-risk Chinese PAH patients.

Although parenteral prostacyclin analogs (PGIs) are the suggested treatment for high-risk PAH, there are several limitations for long-term utilization of PGIs, including not only the economic burden, but also the inconvenience of medication and systemic adverse effects (19).

Treprostinil was previously the only commercially available PGI in China, but the drug is not covered by insurance. In this case, patients are often unwilling to continue treatment with treprostinil, predominantly due to economic burden, even though they remain in a high-risk status. In the present study, most patients who transitioned from treprostinil to selexipag continued to improve or at least remained stable without further deterioration in their disease status. Only one patient continued to decline, which treatment with treprostinil did not prevent. Therefore, for high-risk patients who are unable to afford or unwilling to continue parenteral PGIs, selexipag could be a substitute therapy. However, although several case series demonstrated successful transition from parenteral prostacyclins to selexipag after achieving a low-risk status in PAH patients, some cases exhibited a trend of decline in hemodynamic parameters with relatively stable clinical evaluation, especially in those who respond well to parenteral prostacyclins (20–23). As such, we have to pay close attention to this special transition group during routine clinical visit and hemodynamic monitoring is necessary.

In the present study, we also noticed that improvement in risk assessment and right heart remodeling do not occur in parallel. Although right ventricular diameter and TAPSE improved significantly, the trend in decreasing RA area did not reach statistical significance. Several other studies have also demonstrated that changes in right heart structure and function lagged behind NT-proBNP levels and exercise ability (24, 25). A recently published meta-analysis indicates that the improvement in RV systolic function appeared as long as 6 months and in the right atrial area, 12 months, after initiation of targeted therapy in PAH patients (26).

*Post-hoc* subgroup analysis of data from the GRIPHON trial showed that patients with WHO FC II or III symptoms at baseline similarly benefit from sequential combination of selexipag to background combination therapy with an ERA and PDE-5i (27). Although we only performed descriptive analysis, we also noticed that the WHO FC status between selexipag responders and non-responders was comparable. Recently published retrospective data from French Pulmonary Hypertension Registry illustrate that initiating triple-combination therapy at diagnosis seems to be associated with a higher survival rate in PAH (28). Exploratory analysis of the TRITON study also revealed a trend toward long-term outcome improvement in the initial triple combination treatment group (29). In the present study, patients with shorter PAH history, initial triple combination treatment and relatively minor disease severity, characterized by lower NT-proBNP levels and smaller RAA (30), tended to respond better to selexipag. It is also intriguing that IPAH is the predominant etiology in the responder group. We therefore propose that IPAH patients tend to have a better outcome than post-operative CHD-PAH patients with the same treatment in China.

There are several limitations to our study. First, this is an observational study with limited subjects from a single center, and the follow-up duration was not long enough. Second, invasive homodynamic parameters were not included for analysis. Although hemodynamic risk assessment criteria are independent predictors of transplant-free survival in PAH patients, repetitive RHC procedure is invasive and results in extra economic burden since it is only partially covered by insurance in China. In case that non-invasive risk assessment was already proved useful in identifying patients at low risk (13) and all patients have had at least one RHC before enrollment, only non-invasive parameters were used in the current case series.

## Data availability statement

The original contributions presented in this study are included in the article/Supplementary material, further inquiries can be directed to the corresponding author.

## Ethics statement

The studies involving human participants were reviewed and approved by the Institutional Human Ethics Committee of Qilu Hospital. Written informed consent for participation was not required for this study in accordance with the national legislation and the institutional requirements.

## Author contributions

XC and QJ conceived and drafted the manuscript. WL, DZ, XL, and HuL performed the data collection and interpretation. LQ and HaL validated the data and formal analysis. QJ approved the manuscript. All authors contributed to the article and approved the submitted version.

## Supplementary material

Supplementary Figure 1 Event-free survival in selexipag-treated Chinese PAH patients. Kaplan–Meier curve for time from selexipag initiation to first hospitalization or death event associated with PAH up to the cutoff date of 31 December 2021. Event-free survival of patients in the efficacy set. Patients with PAH disease course >3 years and disease course ≤3 years were calculated separately.
