# Supplementary figures and images for: Selexipag-based triple combination therapy improves prognosis in Chinese pulmonary arterial hypertension patients
Source: Front Cardiovasc Med. 2022 Sep 20;9:991586. doi: 10.3389/fcvm.2022.991586 (PMC9530145; doi:10.3389/fcvm.2022.991586)

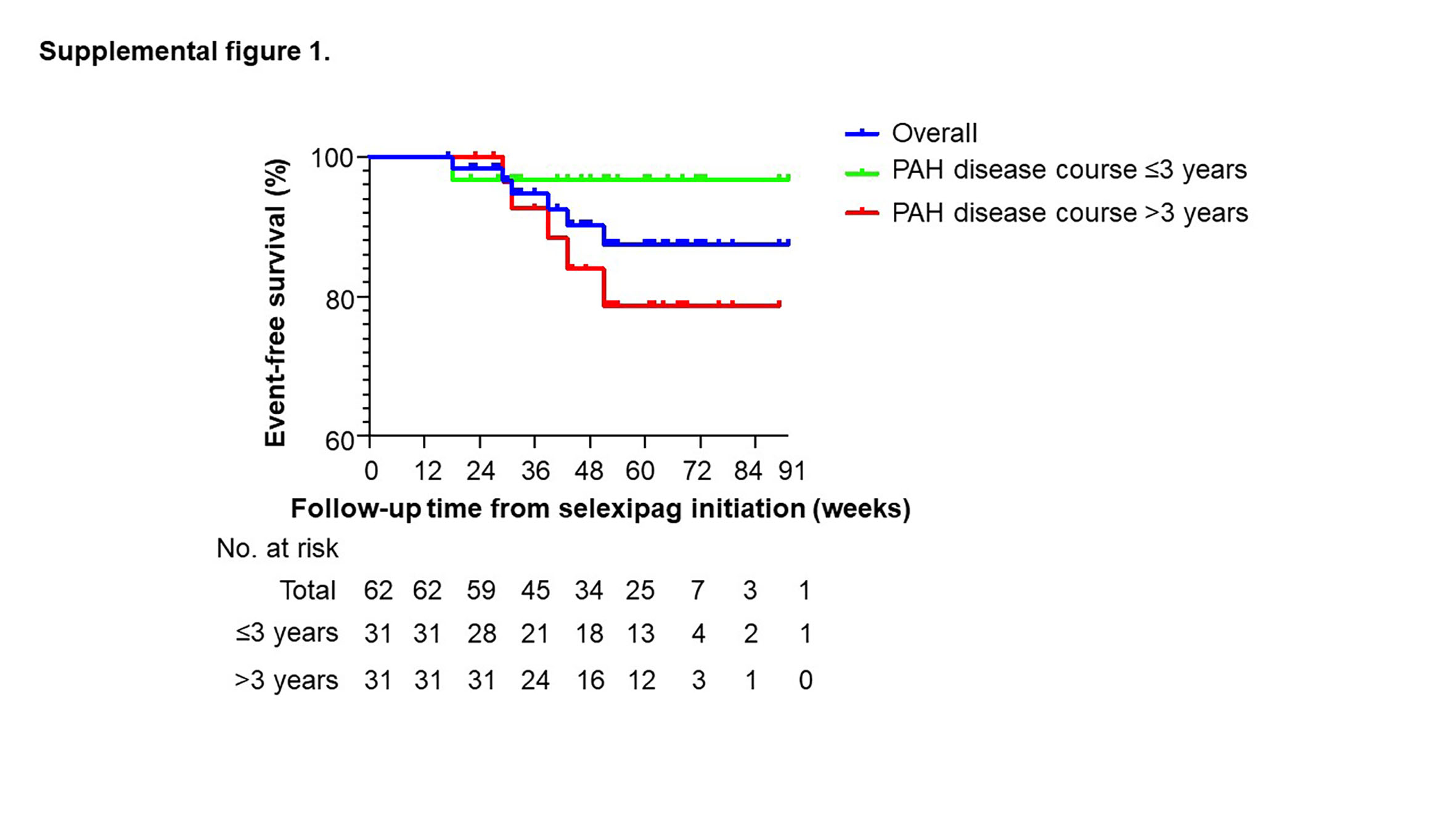

Supplement: Supplementary Figure 1 — Event-free survival in selexipag-treated Chinese PAH patients. Kaplan–Meier curve for time from selexipag initiation to first hospitalization or death event associated with PAH up to the cutoff date of 31 December 2021. Event-free survival of patients in the efficacy set. Patients with PAH disease course >3 years and disease course ≤3 years were calculated separately. [file Image_1.JPEG]
